# S11-5 Extending co-creation in physical activity and health promotion: The Practice Dive approach

**DOI:** 10.1093/eurpub/ckac093.059

**Published:** 2022-08-29

**Authors:** Johanna Popp

**Affiliations:** Department of Sport Science and Sport, FAU Erlangen-Nürnberg, Erlangen, Germany

**Keywords:** Co-production, cooperation, cooperative planning, participation, physical activity

## Abstract

**Background:**

Recently, there has been an increasing interest in co-creation approaches in physical activity (PA) and health promotion. Co-creation involves researchers ?moving beyond the ivory tower? by collaborating with policymakers and practitioners to conjointly develop interventions, e.g. using a Cooperative Planning approach. In addition to the frequently reported benefits of cocreation, there is a growing number of critical voices that highlight the associated challenges, e.g. differing interests and perspectives that inhibit interaction. So far, research has not identified concrete solutions to these problems. This presentation introduces the Practice Dive approach as a potential way of improving cooperation between researchers and practitioners.

**Methods:**

We observed and systematically described a real-life phenomenon in a German research project, which saw project researchers actively moving into practice to familiarize themselves with the setting and the target group (ranging, e.g., from guided factory tours in a manufacturing plant to working at the assembly line for a limited time). We conducted a literature search on similar and related phenomena in co-creational processes and subsequently developed on our observations and findings into a comprehensive concept.

**Results:**

The Practice Dive approach assumes that a significant contribution to a better cooperation between co-creators is the immersion of researchers in the living/working environment of the target group at the beginning of a co-creation process. A stage model can be used to characterize the type and intensity of exchange within the setting and with the target group. Several requirements can be outlined for a successful Practice Dive on the side of the involved co-creators and the setting, including openness and communication skills. Regarding potential effects, different beneficial effects for both researchers and the practitioners can be hypothesized at both the knowledge and the socioemotional level (e.g. familiarity with contextual structures, perceived flatter hierarchies).

**Conclusions:**

The Practice Dive approach has the potential to strengthen the cooperation between research and practice in the field of PA and health promotion and may thus improve the effectiveness of co-creation approaches such as Cooperative Planning. Future research should be aimed at empirically validating the concept and its postulated effects.

